# Patient safety, cost-effectiveness, and quality of life: reduction of delirium risk and postoperative cognitive dysfunction after elective procedures in older adults—study protocol for a stepped-wedge cluster randomized trial (PAWEL Study)

**DOI:** 10.1186/s13063-018-3148-8

**Published:** 2019-01-21

**Authors:** Alba Sánchez, Christine Thomas, Friederike Deeken, Sören Wagner, Stefan Klöppel, Felix Kentischer, Christine A. F. von Arnim, Michael Denkinger, Lars O. Conzelmann, Janine Biermann-Stallwitz, Stefanie Joos, Heidrun Sturm, Brigitte Metz, Ramona Auer, Yoanna Skrobik, Gerhard W. Eschweiler, Michael A. Rapp, PAWEL Study group, Florian Metzger, Andreas Straub, Tobias Krüger, Felix Bausenhart, Petra Renz, Andreas Walther, Carola Bruns, Juliane Spank, Patricia Sabbah, Andreas Häusler, Bernd Förstner, Susanne Schulze, Christoph Maurer, Markus Martin, Bernhard Heimbach, Sebastian Voigt-Radloff, Heiko Reichel, Andreas Liebold, Olivia Küster, Simone Brefka, Stephan Kirschner, Nina Stober, Uwe Mehlhorn, Jürgen Wasem, Anja Neumann

**Affiliations:** 1 Department of Social and Preventive Medicine, University of Potsdam, Am Neuen Palais 10, 14469 Potsdam, Germany; 2 Department of Old Age Psychiatry and Psychotherapy, Klinikum Stuttgart, Stuttgart, Germany; 3 Department of Anaesthesiology and Intensive Care, Katharinenhospital, Klinikum Stuttgart, Stuttgart, Germany; 4 Center for Geriatrics and Gerontology, University Medical Center Freiburg, Freiburg, Germany; 5 University Hospital of Old Age Psychiatry, University of Bern, Bern, Switzerland; 6 Department of Surgery, Medical Center-University of Freiburg, Freiburg, Germany; 7 Department of Neurology, University Hospital Ulm, Ulm, Germany; 8 Agaplesion Bethesda Clinic, Geriatric Center Ulm University, Ulm, Germany; 9 Helios Clinic for Heart Surgery, Karlsruhe, Germany; 10 Institute for Health Care Management and Research, University Duisburg-Essen, Essen, Germany; 11 Institute for General Practice and Interprofessional Care, University Hospital Tübingen, Tübingen, Germany; 12 Geriatric Center Karlsruhe, ViDia Christian Clinics Karlsruhe, Karlsruhe, Germany; 13 Allgemeine Ortskrankenkasse (AOK) Baden-Württemberg, Stuttgart, Germany; 14 Department of Medicine, McGill University Health Center, Glen Campus, Montreal, QC Canada; 15 Geriatric Center at the University Hospital Tübingen, Tübingen, Germany

**Keywords:** Cross-sectoral care, Delirium prevention, Postoperative cognitive dysfunction, Dementia, Older patients, Elective surgery, Quality of life, Cost-effectiveness

## Abstract

**Background:**

Postoperative delirium is a common disorder in older adults that is associated with higher morbidity and mortality, prolonged cognitive impairment, development of dementia, higher institutionalization rates, and rising healthcare costs. The probability of delirium after surgery increases with patients’ age, with pre-existing cognitive impairment, and with comorbidities, and its diagnosis and treatment is dependent on the knowledge of diagnostic criteria, risk factors, and treatment options of the medical staff. In this study, we will investigate whether a cross-sectoral and multimodal intervention for preventing delirium can reduce the prevalence of delirium and postoperative cognitive decline (POCD) in patients older than 70 years undergoing elective surgery. Additionally, we will analyze whether the intervention is cost-effective.

**Methods:**

The study will be conducted at five medical centers (with two or three surgical departments each) in the southwest of Germany. The study employs a stepped-wedge design with cluster randomization of the medical centers. Measurements are performed at six consecutive points: preadmission, preoperative, and postoperative with daily delirium screening up to day 7 and POCD evaluations at 2, 6, and 12 months after surgery. Recruitment goals are to enroll 1500 patients older than 70 years undergoing elective operative procedures (cardiac, thoracic, vascular, proximal big joints and spine, genitourinary, gastrointestinal, and general elective surgery procedures).

**Discussion:**

Results of the trial should form the basis of future standards for preventing delirium and POCD in surgical wards. Key aims are the improvement of patient safety and quality of life, as well as the reduction of the long-term risk of conversion to dementia. Furthermore, from an economic perspective, we expect benefits and decreased costs for hospitals, patients, and healthcare insurances.

**Trial registration:**

German Clinical Trials Register, DRKS00013311. Registered on 10 November 2017.

**Electronic supplementary material:**

The online version of this article (10.1186/s13063-018-3148-8) contains supplementary material, which is available to authorized users.

## Background

Delirium is associated with increased morbidity and mortality, cognitive impairment, dementia, and higher institutionalization rates [1]. The incidence of postoperative delirium (POD) depends on factors predisposing to delirium such as age, the presence of brain damage, dementia, deficits in cognitive, sensory, or mobility functions, multiple comorbidities, polypharmacy, and frailty [2]. Early indicators of cognitive deficits, including hyposmia [3], sleep disorders [4, 5], and subjective memory impairment [6, 7], are also relevant risk factors for delirium. The perioperative phase is a major trigger of postoperative delirium because of the administration of anesthesia, the surgical procedures, and other factors related to the operation itself, such as pain and immunological activation [2]. Postoperative cognitive dysfunction (POCD) often appears after POD [8, 9] and has been studied in relation to the preoperative status in order to measure the delirium’s impact, particularly on the risk of developing dementia [10], and in order to estimate the related health costs. POD and POCD are associated with higher mortality and postoperative complications (such as infections, falls, decubitus ulcers, incontinence) [9], with prolonged hospital stays [11], with the need for longer/extended intensive care and therapy and higher nursing workload burden [12], and with increased costs for both hospitals and healthcare insurance providers [2, 13].

Delirium is a healthcare quality indicator in older adults, and therefore delirium prevention is an essential parameter for patients’ safety [14, 15]. The incidence of delirium and its severity and duration can be significantly decreased and may be stratified by taking delirium risk factors into account [16]. Current guidelines for POD management [17] emphasize the importance of delirium prevention. A multimodal nonpharmacological approach [14, 18] is considered the best pathway [16, 19]. In a meta-analysis [20], this approach reduced the delirium risk by 53% (95% CI, 0.38 to 0.58) when comparing intervention and control groups.

For elective surgery, it is possible to implement a preadmission delirium prevention plan [21], adapting care to the patient’s age and delirium risk. However, surgical centers typically do not implement preadmission procedures for delirium risk assessment, and recommended interventions for the management of delirium are not standardized.

In this study, we will develop a cross-sectoral and “best practice” multimodal delirium prevention approach in five medical centers. The proposed multisectoral model for delirium prevention integrates and builds upon several multimodal admission models [16] with preadmission risk reduction counseling (“prehabilitation”), perioperative monitoring [22], and training based on international guides [17, 23] of multidisciplinary patient care providers, including operation room personnel, service staff, and families. The study will optimize delirium assessment, establish a cross-sectoral intervention bundle for preventing delirium, and evaluate the effectiveness and cost-efficiency of this all-encompassing approach. To this end, evidence-based delirium diagnoses, neuropsychological tests, and multimodal multiprofessional interventions will be implemented. Follow-up POCD evaluations will be performed 2, 6, and 12 months after surgery. Furthermore, relatives will be asked about their care burden, and a cross-sectoral analysis of the perioperative patient pathway will be performed.

The project, recruiting a total of 1500 patients, has the following objectives:

- The evaluation of the perioperative delirium prevalence with the I-Confusion Assessment Method-based scoring system for delirium diagnosis and delirium severity (I-CAM-S) [9], which is used for the first time in a large multicenter German sample.
- The implementation of a multisector, individualized, multiprofessional and multimodal delirium and POCD prevention program.
- The evaluation of the prevalence of POCD at 2 and 6 months after surgery, and the persistence of POCD after 12 months.
- The evaluation of changes in medication during the pre-admission and perioperative phases, especially with respect to avoidance of anticholinergic drugs and other pharmacologic agents associated with delirium.
- The evaluation of the care burden of the patients’ relatives.
- The economic evaluation of the multimodal intervention, studying its cost-effectiveness. From the point of view of the hospital departments, initial hospital costs will be evaluated. From the point of view of the health and care insurance company Allgemeine Ortskrankenkasse (AOK) Baden-Württemberg (Germany), inpatient and outpatient costs during the 12 months before and after the surgery will be analyzed in relation to outcome differences.

## Methods

### Design

**Fig. 1:**
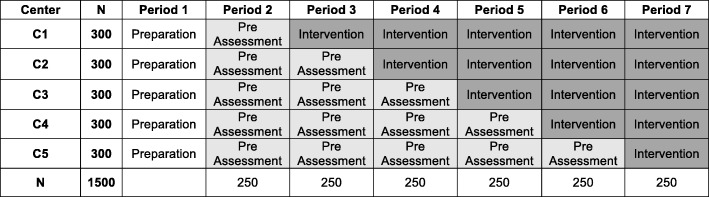
Timeline and randomization

We have designed a cross-sectoral longitudinal study that aims to include 1500 patients undergoing elective surgery. The study employs a stepped-wedge design with cluster randomization of five medical centers. The study will have seven periods, each lasting 12 weeks (see Fig. 1). The study will evaluate an all-encompassing “best practice” multimodal intervention for preventing delirium and POCD that includes six consecutive measurement points: preadmission, preoperative, and postoperative including daily delirium screening for 7 days after surgery and 2, 6, and 12 months after surgery. We aim to show that: the cross-sectoral multimodal and multidisciplinary intervention reduces the delirium rate by at least 40% compared with treatment as usual; the cross-sectoral multimodal and multidisciplinary intervention reduces the rate of postoperative cognitive decline by at least 20% compared with treatment as usual; and the cross-sectoral intervention, including the team training and the modules for nonpharmacological prevention, is cost-effective and therefore the improvement in quality of life imposes no additional costs because the care needs of the patients and caregivers are lower than with the standard treatment.

The study strategy is registered, constructed, and presented according to the recommendations of the Standard Protocol Items: Recommendations for Interventional Trials (SPIRIT) [24, 25] (see Additional file 1: SPIRIT checklist). The items from the World Health Organization Trial Registration Data Set are presented in Table 1.

**Table 1:** Items from the World Health Organization Trial Registration Data Set

1	Primary registry and trial-identifying number: DRKS-ID, DRKS00013311
2	Date of registration in primary registry: 10 November 2017
3	Secondary identifying numbers: no
4	Sources of monetary or material support: Innovation Fund of the Federal Joint Committee (G-BA): VF16012
5	Primary sponsor: University Hospital Tübingen
6	Secondary sponsor(s): (to be found)
7	Contact for public queries: Prof. Dr med. Gerhard Eschweiler, Geriatric Center of the University Hospital Tübingen; PD. Dr med. Christine Thomas, Klinikum Stuttgart
8	Contact for scientific queries: Prof. Dr med. Dr phil. Michael Rapp, Social and Preventive Medicine, Potsdam University
9	Public title: PAWEL: Patient safety, cost-effectiveness and quality of life: reduction of delirium risk and post-operative cognitive dysfunction after elective procedures in the elderly
10	Scientific title: see 9
11	Countries of recruitment: Germany, Baden-Wuerttemberg
12	Health condition(s) or problem(s) studied: delirium, postoperative cognitive dysfunction (POCD)
13	Intervention(s): trans-sectoral multimodal perioperative intervention for elective surgical interventions vs treatment as usual (TAU)
14	Key inclusion criteria: patients older than 70 years undergoing an elective surgery (heart, thorax, vessels, proximal big joints and spinal cord, genitourinary, gastrointestinal, and general elective surgery procedures) with at least 60-min duration of anesthesia (cut-to-suture time) Key exclusion criteria: emergency surgery, newly discovered severe dementia (red flag: Mini Mental State Examination (MMSE) < 15, Montreal Cognitive Assessment (MoCA) < 8) without caregiver holding power of attorney, 120 km of driving distance to the center, inability to consent due to decreased German language abilities, poor clinical prognosis (survival < 15 months)
15	Study type: stepped wedge cluster randomized design
16	Date of first enrolment: 20 November 2017
17	Target sample size: 1800 for the delirium risk score, 1500 thereof for comparison of intervention
18	Recruitment status: enrolling by invitation
19	Primary outcome(s): delirium prevalence, measured by daily delirium screening (I-Confusion Assessment Method-based scoring system for delirium severity (I-CAM-S)) over 7 days post surgery, as well as after 2 and 6 months; Nursing Delirium Screening Scale (NuDESC) on days 2 and 6 post surgery
20	Key secondary outcome(s): delirium duration as described in the primary outcome assessment. Prevalence of POCD 2 and 6 months after surgery as measured by a neuropsychological test battery (Montreal Cognitive Assessment (MoCA), digit span backwards and Trail Making Test A and B (TMT A and B)) as well as cognitive performance measured with the continuous nonstandardized test values of these scales. A cognitive deficit is defined as the presence of a test value ≤ 0.5 standard deviations, normalized for age, gender, and education, in one of these test procedures

### Trial overview

The consortium leader will manage the project, oversee the financial transfers, monitor the progress according to the planned schedule, and communicate with the study sponsor Innovationsfonds des Gemeinsamen Bundesausschusses. The consortium leader is also part of the steering committee, whose main tasks are dealing promptly with the everyday project issues, monitoring the recruitment progress and the implementation of the intervention modules, and managing the study documentation. The members of the steering committee are also part of the project committee, which includes two members from each study site. The project committee accompanies the study and coordinates the joint publications as well as the requests for data analysis. If necessary, the project committee, based on interim analyses performed every 3 months, can decide on early termination of the project. Moreover, in its meetings every 6 months, the committee may deal with any relevant project issues. An international scientific advisory board of well-established professionals, including a geriatrician, an anesthetist, a gerontologist, and a delirium expert, ensures the scientific quality of the trial. The advisory board is comprised of four delirium and POCD experts. An external Regional Ethics and Data Monitoring Board (REDMB) includes national experts on delirium and POCD who are independent of the sponsor and trial investigators, and have no competing interests. They may be called upon to deal with ethically difficult issues, and they will also act as a monitoring board for adverse events (AEs). Furthermore, if the trial was to terminate early, the REDMB would take part in that decision.

### Participants

#### Inclusion criteria

Eligible patients are aged 70 years or older and scheduled for elective surgery (cardiac, thorax, vessels, proximal large joints or spine, genitourinary, abdominal, or general elective surgery procedures) with a planned duration of surgery of at least 60 min (cut-to-suture time) under general, spinal, or regional anesthesia. As delirium is a high risk in dementia and frailty, we include patients with dementia or frailty who can consent to the trial or whose substitute decision-makers provide consent. 

#### Exclusion criteria

Patients undergoing emergency surgery procedures, patients unable to consent due to insufficient mastery of the German language or with newly discovered severe dementia (red flag: Mini Mental State Examination (MMSE) < 15, Montreal Cognitive Assessment (MoCA) < 8) without a substitute decision-maker, patients with a poor clinical prognosis (expected survival of less than 15 months), and patients who have a long driving distance to the study site (> 120 km) are to be excluded from the trial.

**Fig. 2:**
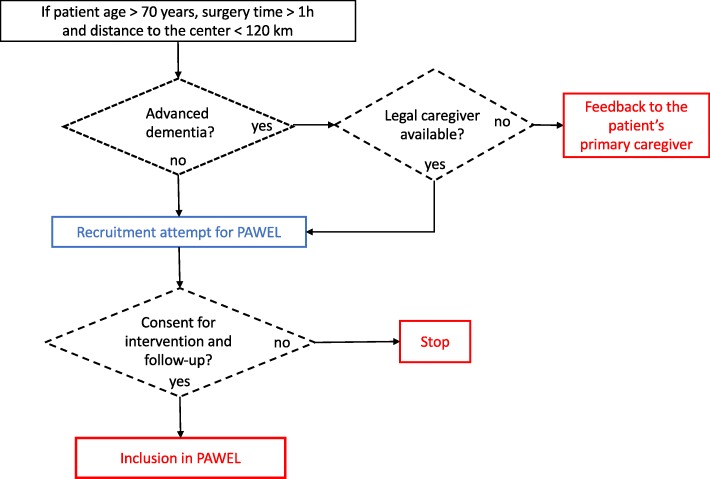
Recruitment procedure. PAWEL Patient safety, cost-effectiveness and quality of life: reduction of delirium risk and post-operative cognitive dysfunction after elective procedures in the elderly

The recruitment procedure is described in Fig. 2. Patients who are not able to consent may also be recruited if the legal guardian consents to the patient’s study participation, given that such patients are especially at risk of developing delirium and POCD after surgery [10], and they could eminently benefit from the intervention.

#### Intervention

The intervention implements a cross-sectoral all-encompassing multimodal delirium prevention and management approach, and will be carried out in each study center after the center is randomized to take part in the intervention phase of the trial:

1. All personnel training: within 4–6 weeks before the start of the intervention phase, nurses and therapeutic, medical, and support staff involved in the care of study patients will be trained with respect to dementia and delirium care, delirium diagnosis, and depression using a standardized training plan. Staff will be trained to three different levels of expertise: more than 70% of the staff are to receive basic training (90-min training time); more than 20% of nurses are to receive advanced training (“delirium scout” training—450-min training time); and more than 10% are to receive expert-level training (“delirium champion” training—900-min training time). Additionally, at least 70% of the physicians will receive 90 min extra of specific training about delirium risk, diagnosis, treatment, and prevention. The courses will be adjusted to the specific surgical and anesthesiological departments and special care units in order to ensure comparable levels of knowledge between different study sites.
2. Environmental orientation support: the hospital environment will be adapted to the special needs of the patients. The decline of sensory function in older adults often leads to additional psychosocial stress, and is exacerbated by cognitive impairment. To address these issues, appropriate posters and signage will be placed on the wards, and in patients’ rooms and restrooms. Tools for temporal and situational orientation will be made available; for example, whiteboards with personal information, date, season, and year, as well as analog clocks that can be seen from the bed. Moreover, appropriate tools to prevent falls will be provided, such as anti-sliding socks. Special boxes for glasses, hearing aids, and dentures, as well as sleeping masks and ear plugs will be within reach of the patients at all times.
3. Preadmission phase: the trained staff will implement some nonpharmacological preventive interventions in this phase, including informative talks and written recommendations for patients, and instructive contacts with the referring doctors regarding the age-appropriateness of prescribed medications and interventions.
4. “Best practice” multimodal intervention: participants at the respective centers will receive perioperative and postoperative multimodal delirium prevention and care management between 8 a.m. and 8 p.m. on 7 days of the week, modified according to best practice models such as the Hospital Elder Life Program (HELP) [26], “The old patient in the surgery room” [22], or the Care of Confused Hospitalised Older Persons (CHOPs) [27]. Psychogeriatric nurses and one physician who have received 900 additional minutes of “delirium champion” training will form a multiprofessional consultation–liaison-like intervention team that will contact the patients and address their special needs daily. They will facilitate the implementation of the treatments advised in international delirium prevention guidelines [17, 23]: adaptation of surgery and anesthesia, medication and pain treatment appropriate for the patients’ age, pain monitoring, avoidance of movement restrictions such as catheters or infusions, and avoidance of benzodiazepines and anticholinergic drugs. These specialists will prescribe meaningful individualized daily activities for preventing delirium, defined in six modules: reorientation, cognitive activation, mobilization, meal companionship, clinical diagnostics and operation room attendance, and nonpharmacological sleep promotion and anxiety reduction. A team of nurses’ aides or volunteers (mostly gap-year social work volunteers) will carry out these activities, as well as chaperone patients during the diagnostic procedures, and in the operating and recovery rooms. They will receive 40 h of theoretical and practical training on the modules, and will be available daily in two shifts of 12 h per day.
5. Patients and their family members will be advised individually about delirium risk and prevention, and will receive information materials (leaflets, posters, etc.) about delirium and the care service. Moreover, family members will provide individual information about the patient, facilitating individualized care and communication, and will be advised to support some individualized delirium prevention activities.

Given the patient-centered care research implementation goal of this study, the outcomes of patients undergoing the intervention will be compared to the outcomes of patients receiving treatment as usual (TAU) provided by the centers before the randomized start of the intervention.

Patient group involvement and staff involvement has been implemented in an earlier version of this intervention approach adapting the HELP (Hospital Elderly Life Program) structure (see [18]). In addition, we will implement focus groups at the general practitioner level post hospital stay to further evaluate patients’ perception of the intervention itself.

#### Adherence to intervention

The training modules are expected to play a fundamental role in improving adherence to the intervention protocols. They will be supplemented by additional talks, case discussions, and a web-based knowledge base providing webinars, training videos, and so forth. Moreover, a support system for the intervention teams will be established comprising an email service and a telephone hotline, as well as a data pool on a project server. Modules prescribed by the intervention team will be monitored, and the time span of the intervention will be daily documented. Furthermore, adherence to the manuals and “prescription checklists” will be tested by unannounced visits to every site.

#### Adverse events

Adverse events and serious adverse events (SAEs) will be recorded and documented. Falls, strokes, infections, and other severe perioperative complications (death, reoperation, pneumonia, sepsis) are to be expected in this patient group independently of the intervention, while SAEs related to the intervention are expected to be very rare. An example of a study-related SAE could be, for instance, a patient’s fall during early mobilization by the delirium companion during the active intervention phase. The feasibility of the project and the occurrence of SAEs will be assessed every 3 months by the REDMB, who will also carry out audits every year. In the case of substantial differences between the SAEs in the different groups, this will be discussed by the REDBM.

#### Withdrawal

The criteria for discontinuing the intervention for a participant will be death; or study withdrawal requested by the patient, guardian, or authorized relative; or (re)operation of more than 1 h (cut-to-suture time) during the first perioperative week, given that in this case a delirium or POCD cannot be precisely assigned to the first operation; or malignancy surgery with prognosis under 15 months, neoadjuvant chemotherapy, brain radiotherapy, or primary metastasis surgery for pancreas or bronchial cancer.

#### Outcome assessment

The assessment of all the outcomes will always be performed by trained assessors, who will be blinded for the intervention. Specifically, delirium raters will be told that the data will be used for validating a delirium risk score. Staff will be instructed not to reveal the nature of the intervention to these assessors.

The *primary outcome* will be delirium prevalence. It will be measured by daily delirium screening (I-Confusion Assessment Method-based scoring system for delirium severity (I-CAM)/CAM-S)) [28, 29] over 7 days after surgery and after 2 and 6 months, the Nursing Delirium Screening Scale (NuDESC) [30] (days 2 and 6 after surgery), a chart review at discharge applying the DSM-V delirium criteria as a reference standard [31], and the clinical evaluation.

The CAM [32], with its four-step diagnostic algorithm, is a widely used screening test for assessing delirium. Originally developed from the DSM-III-R, it is now predominantly used for delirium screening and research according to the DSM-IV and DSM-V criteria. It has been operationalized and translated into German [33], and then revealed a high sensitivity of 0.77 in a cohort of geriatric patients with a high prevalence dementia, and a specificity of 0.96–1.00 with excellent inter-rater reliability (Cohen’s κ = 0.95 (CI 0.74–1.0) for the algorithm, single item’s κ values varied between 0.5 and 1).

The I-CAM (I for ICD-10) [28] extends the German version of the original CAM adding abnormal psychomotor activity, to allow taking also the ICD-10 delirium diagnosis as a reference standard and assessing the motor delirium subtypes as well. The CAM-S [29] is a CAM-based scoring system for assessing delirium severity and was operationalized for use with the German version of the I-CAM. As the CAM might be confounded by the fluctuating nature of delirium, we use a chart-based review [31] filled out by trained medical staff at discharge to evaluate for fluctuations in sleep–wake rhythm or psychomotor activity indicating delirium.

The NuDESC is a five-item scale based on nurses’ observations assessing disorientation, inappropriate behavior and communication, hallucinations, and psychomotor retardation over a 24-h period. For the German version of the NuDESC, in a sample of patients after elective surgery, a sensitivity of 0.98, a specificity of 0.92, and an inter-rater reliability of 0.83 were observed [34].

The *secondary outcomes* will be: delirium duration as described in the primary outcome assessment; prevalence of POCD 2 and 6 months after surgery; and persistence of POCD after 12 months. The prevalence of POCD will be measured by the following neuropsychological test battery: the Montreal Cognitive Assessment (MoCA) [35], the digit span backwards [36], the Trail Making Test A and B (TMT A and B) [37], and cognitive performance measured with the continuous nonstandardized test values of these scales. A cognitive deficit is defined as the presence of a test value of ≤ 0.5 standard deviations, normalized for age, gender, and education, in one of these test procedures.

The MoCA is a brief cognitive screening test for assessing cognitive impairment among older people. The test assesses multiple cognitive domains including visuospatial ability, executive functions, memory, attention, language, abstraction, and orientation. The MoCA has high sensitivity (0.90) and specificity (0.87) to detect individuals with mild cognitive impairment and distinguish them from cognitively intact older people [35] and is available in three parallel versions.

The digit span backwards is commonly used to assess working memory capacity. Participants are required to recall a sequence of spoken digits in reverse order. For people older than 70 years, the digit span backwards had a test–retest reliability > 0.60 and an internal consistency of 0.882 [38].

The TMT is a widely used instrument in neuropsychological assessment that measures the speed of scanning and visuomotor tracking, divided attention, and cognitive flexibility [36]. The test consists of two parts, A and B. TMT A requires an individual to draw lines sequentially connecting consecutive numbers from 1 to 25. TMT B involves drawing a similar line, connecting an ascending sequence of numbers and letters in an alternating manner. In a sample of healthy older adults, Part A had a test–retest reliability of 0.78 and Part B of 0.73, and Part A an inter-rater reliability of 0.99 and Part B of 0.93 [39]. In a sample of elderly volunteers, Part B had a sensitivity of 0.63 for cognitive dysfunction, 0.72 for dementia, and 0.77 for AD, and a specificity of 0.89 [40].

For *baseline assessment*, the following variables will be evaluated. Basic sociodemographic patient information to be collected includes age, gender, weight, height, dominant hand, marital status, immigrant background, educational level, occupation, living arrangements, nicotine consumption, alcohol consumption, falls, and statutory level of care dependency. The self-reported subjective memory impairment (SMI) will be assessed, for which subjects will be asked “Do you feel that your memory is getting worse?” (no; yes; I don’t know). If the patient answers yes, the patient will then be asked whether he or she is worried about this (no; yes, that worries me; yes, that worries me very much; I don’t know, no answer) [41]. The personal medical history, including comorbidities, is to be quantified by the expanded version of the Charlson’s comorbidity Index (CCI) [42]. The patient’s preoperative physical status will be assessed by the American Society of Anesthesiologists Physical Status classification (ASA) [43]. The risk of stroke will be estimated with the CHA_2_DS_2_-VASc score (Congestive heart failure/left ventricular dysfunction, Hypertension, Age > 75, Diabetes, Stroke/transient ischemic attack/thromboembolism, Vascular disease, Age 65–74, Sex category) [44]. Laboratory results (hemoglobin, sodium, creatinine, total amount of protein, C-reactive protein), the history of delirium in the past, and a neurological examination will be recorded. The grip strength as a frailty marker will be measured with the Jamar® Hydraulic Hand Dynamometer. Hearing and visual integrity will be tested by the whisper and visual acuity tests [45]. Functional mobility and risk of falls will be investigated by the Timed Up and Go Test (TUG) [46]. Anxiety and depression will be assessed by the Patient Health Questionnaire (PHQ-4) [47]. The health-related quality of life will be measured by the EuroQol five dimensions questionnaire (EQ-5D-5 L) [48, 49] (this version includes five levels of severity in each of the existing five EQ-5D dimensions) and by the 12-Item Short Form Survey (SF-12) [50, 51]. Nutritional status will be assessed by the Mini Nutritional Assessment Short Form (MNA-SF) [52, 53]. Functional status will be evaluated with the Hamburg Classification Manual [54] version of the Barthel Index [55]. Frailty will be analyzed with the Clinical Frailty Scale of the Canadian Study of Health and Aging (CSHA Clinical Frailty Scale) [56]. Pain will be measured by the Numerical Rating Scale of Pain (NRS Pain) [57]. Sleeping behavior will be investigated by the Pittsburgh Sleep Quality Index—Basic (PSQI-Basic), a four-item version of the PSQI [58]. Sleep apnea will be screened for by the STOP-BANG questionnaire (Stop-BANG) [59]. Olfactory function will be scrutinized by the Sniffin’ Sticks 12 version [60]. Cognitive decline will be assessed by the very short version (seven items) of the Informant Questionnaire on Cognitive Decline in the Elderly (IQCODE) [61]. The subjective burden of the family caregivers will be measured by the German Zarit Burden interview (G-ZBI) [62, 63]. Classification as a geriatric patient, will be assessed with the Geriatric-Check using the geriatric concept of Baden-Württemberg (GC) [45]. Finally, the patient’s medications will be recorded, including the type, dose, and frequency of administration.

**Fig. 3:**
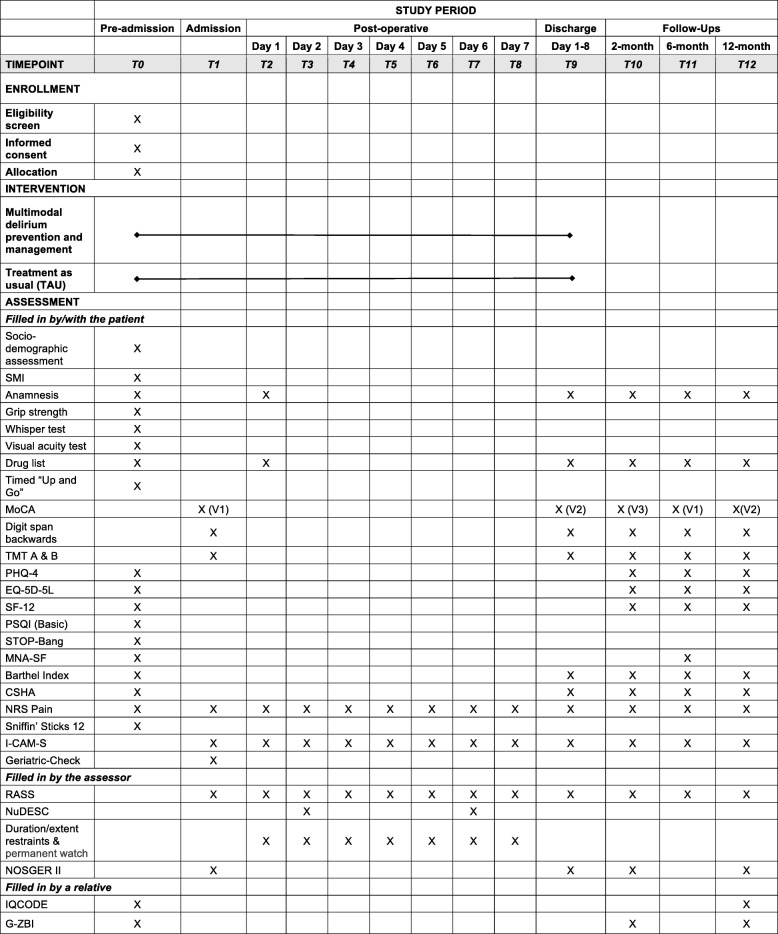
Standard Protocol Items: Recommendations for Interventional Trials figure of enrollment, intervention, and assessments. CSHA Clinical Frailty Scale of the Canadian Study of Health and Aging, EQ-5D-5L EuroQol five dimensions questionnaire, G-ZBI German Zarit Burden interview, I-CAM-S I-Confusion Assessment Method-based scoring system for delirium severity, IQCODE Informant Questionnaire on Cognitive Decline in the Elderly, MNA-SF Mini Nutritional Assessment Short Form, MoCA Montreal-Cognitive Assessment, NOSGER II Nurses’ Observation Scale for Geriatric Patients, NRS Pain Numerical Rating Scale of Pain, NuDESC Nursing Delirium Screening Scale, PHQ-4 Patient Health Questionnaire, PSQI (Basic) Pittsburgh Sleep Quality Index (Basic), RASS Richmond Agitation–Sedation Scale, SF-12 12-Item Short Form Survey, SMI subjective memory impairment, TMT Trail Making Test, V1/V2/V3 parallel versions of the MoCA, STOP BANG Sleep Apnea Questionnaire (snoring, tiredness, observed apnea, blood presure, body mass index, age, neck circunference, gender)

For *planned analysis*, the following variables are currently anticipated: anxiety and depression will be evaluated with the PHQ-4 questionnaire, the health-related quality of life with the EQ-5D-5 L questionnaire and the SF-12 survey, the nutrition status with the MNA-SF, the functional status with the Barthel Index, and the frailty with the CSHA Clinical Frailty Scale and hand grip. The duration and extent of use of physical restraints and patient care attendants will also be assessed. Moreover, the relevant aspects of behavior in daily life will be assessed by the Nurses’ Observation Scale for Geriatric Patients (NOSGER II) [64, 65], the presence of cognitive decline will be assessed by IQCODE evidenced by relatives, and the subjective burden of family caregivers by G-ZBI. The timelines are summarized in Fig. 3.

For *planned validity assessment of postoperative delirium*, the validity of the I-CAM-S and NuDESC vs the Richmond Agitation-Sedation Scale (RASS) [66] and vs chart review will be evaluated. The RASS is a simple observational scale that quantifies the level of consciousness, assessing both sedation and agitation.

For *analysis of delirium severity*, the duration and extent of restraints and one-to-one supervision requirements will be used, in addition to the CAM-S score.

For *process analysis*, acceptance and feasibility will be evaluated by qualitative assessment using focus groups formed by members of the geriatric centers.

For *planned health-economic analyses*, the initial hospital costs and the costs per delirium of two centers (Tübingen and Stuttgart) will be assessed. Moreover, for the patients whose health insurance is AOK Baden-Württemberg (Germany), health and care insurance expenditures and utilization will be assessed, in particular regarding hospital care, outpatient care, medication, rehabilitation, and nursing care. For a precise evaluation of the costs, the expense for training and staff carrying out the intervention will be included in the analyses.

#### Timelines

The study started in November 2017, and the recruitment phase will last until April 2019.

There will be three follow-up examinations after 2, 6, and 12 months, with the last planned for April 2020. We plan to complete the evaluations and cost analyses by December 2020.

From the patient’s perspective, the study will start with a preadmission screening (T0, at most 3 weeks before admission) (see Fig. 3). Admission (T1) will be followed by surgery and then daily delirium screening for 7 days (T2–T8) during hospitalization. A final discharge assessment (T9) will take place 1–8 days after surgery. If, at this point, the patient is considered to suffer from delirium, then delirium screening and monitoring will continue for another 2 weeks, so long as the patient remains hospitalized in the center. The discharge examination (T9) will take place, at the latest, 3 weeks after the elective surgery, with the participation of a physician who will note any postoperative complications. Follow-up assessments will be performed after 2 months (T10), 6 months (T11), and 12 months (T12). Moreover, the patient’s relatives will be asked at T12 about their care burden using the G-ZBI, and the cognitive state of the patient will be assessed using the IQCODE instrument. If a patient is unable or unwilling to go to the regional center for any follow-up assessment, the local PAWEL team will offer the assessment either at some hospital closer to the patient or at the patient’s general physician’s practice. If the patient is not able to go to any of these places, a team member will assess the patient at the patient’s home. Finally, if the patient refuses this option, he or she will be asked for a telephone interview.

#### Sample size

The power analysis for the primary outcome of delirium prevalence assumes a reduction in the outcome from 25% to 15% [20] as a result of the intervention. A conventional analysis to detect differences in proportions (delirium rate) between the intervention and control groups, using Fisher’s exact test, results in a total of 514 patients with a 1:1 randomization, given a power of 1 – β = 0.80 and an α error of 5%. Using the adjustment form proposed by Woertman et al. [67] with five crosspoints in a stepped-wedge design, with a maximum of 50 patients per cluster per period and an intracluster correlation of 0.01, leads to a correction factor for the stepped-wedge design of KF = 2.63, and therefore a number of 514 × 2.63 = 1351 patients. Assuming a dropout rate of 15%, this leads to a total requirement for about 750 patients per arm. The minimum number of clusters is given by the ratio of the total number of patients to the product between the number of crossing points and the patients per cluster per period, and is 15.

For the secondary outcomes, a reduction of the persistent cognitive deficit from 20% to 10% results in 428 patients using the conventional analysis with Fisher’s exact test and a 1:1 randomization, a power of 1 – β = 0.80, and an α error of 5%, which after the design correction leads to a requirement of 1079 patients.

Thus, the planned sample size is of at most 1500 patients (see Fig. 1 with the numbers per period). Furthermore, relatives of the patients will be asked about the burden of caring and the cognitive status of the patients.

#### Recruitment

The study takes place in Germany in the state of Baden-Württemberg. The study will be performed in university hospitals in Tübingen, Freiburg, and Ulm, and in tertiary care hospitals (one in Stuttgart and two in Karlsruhe). In total, five medical centers (one per city) with 12 surgical departments (three in Stuttgart and Tübingen, and two at the other centers) are included. To balance the surgical subspecialties per center, recruitment is restricted to a maximum of 2/3 from one of the following surgical subspecialties: orthopedics, vessels and cardiac, or abdominal. We intend to maintain this ratio for every center and for every 12-week interval of the study.

Patient recruitment will be carried out by a medical specialist. Study staff, including doctors and scientists, will provide the necessary information and obtain the written informed consent from the patients, and whenever possible also from one relative (to assess the caregiver burden and the cognitive status of the patient).

The medical directors and the managers of anesthesia and nursing at the hospitals included in the study have provided a letter of intent expressing their interest in introducing and evaluating preventive procedures, and their willingness to recruit the necessary number of patients. Hence, the recruiting objective is realistic. However, if a department fails to meet its recruitment target (17 patients for the departments in Stuttgart and Tübingen, 25 patients for the others) during one period, concrete and binding measures will be taken to reach the planned number of patients. In this case, the center will commit either to increase the recruitment in its departments or to include another surgical department. If several departments do not reach their target, then the five recruitment blocks, each lasting 3 months, will be extended to 4 months, adding 33% more time to recruit. In this case, for the patients recruited during the last period of the project, the POCD assessments would be completed after 6 months instead of after 12 months.

#### Randomization

Randomization will take place at the cluster level (*k* = 5 clusters) using a computer-generated sequence. Five months before starting the intervention in a given center, that center and the training team in Stuttgart will be informed about the allocation to prepare for the training and to form the intervention teams. Otherwise, randomization allocation will only be known to the consortium leader and the scientific staff at the University of Potsdam, who will implement the random sequence generation.

#### Data management

The collection of the clinical data during all stages of the study (preadmission, admission and postoperatively) will be performed exclusively by authorized staff of the study sites, using electronic Case Report Forms (eCRFs) via the web-based electronic data capture (EDC) system secuTrial® from interActive Systems GmbH. The access to the secuTrial® platform is protected by an authentication process, and the transmission of data between the study centers and the secuTrial® server is protected by a secure connection using Secure Sockets Layer (SSL) encryption. All collected data will be checked for reliability and validity every 3 months. Data access will be granted initially to project and data management and consortium leaders. All the data from scales and neuropsychological tests, as well as the clinical data and the data for the health-economic analysis, will be stored in a pseudonymized fashion. After the end of the study, all paper and electronic documents will be stored for at least 10 years more.

#### Statistical analysis

For the primary outcome, logistic regression analyses with cluster adjustment are planned.

For the secondary outcomes, the changes in the medication and the care burden of the patients’ relatives, we plan analyses using mixed linear and logistic regression models, with fixed and random factors for estimating heterogeneity over clusters [68].

Subgroup analyses for specific patient groups (e.g., patients with cardiac surgery regarding cognitive status or patients with brief hospital stays/brief surgeries) are planned.

For planned validity assessments of postoperative delirium, the validity of the I-CAM-S and NuDESC vs the RASS will be evaluated as screening tools for postoperative delirium. Diagnostic test performance of the tools will be evaluated by receiver operating characteristics (ROC) analysis.

Missing data (due to nonadherence of a department or a particular patient) will be documented. We will apply mixed-effect models, which handle the missing data without using any imputation method. Mixed-effect models assume that the missing data are missing at random (MAR); that is, when the probability that an outcome is missing is related to some other fully observable variable in the model, but not to the variable with the missing value itself [69].

Regarding the health-economic evaluation, a micro-costing analysis will be carried out using data from the clinical administration of two hospitals (Tübingen and Stuttgart). Moreover, for the patients insured by a specific health insurance (AOK Baden-Württemberg) who consent, the health and care insurance costs of the 12 months before and after surgery will be calculated using a difference-in-difference approach [70]. In this case, the target parameter is the cost per avoided POCD. The cost-effectiveness of the intervention will be calculated comparing the costs of the intervention group and the control group. In addition, quality-adjusted life years (QALYs) will then be calculated using data from the EQ-5D-5 L form about health-related quality of life, and applied for calculating the incremental cost-effectiveness. When possible, subgroup analyses will be carried out, for example, by age or gender, to determine whether cost-effectiveness is different in the corresponding groups. Sensitivity analyses will also be performed. The evaluation will be carried out following the recommendations of the “Good practice secondary data analysis” (GPS).

#### Dissemination policy

Publications about the results of the trial will be submitted to international journals after being approved by the project committee. Use of professional writers will not take place, and authorship eligibility criteria are specified in the trial contract and comply with International Committee of Medical Journal Editors (ICMJE) statements [71].

An analysis of the perioperative patient pathway will be developed as an outcome of the project. The goal of this analysis is to adapt the intervention to everyday care routine and to facilitate its subsequent implementation. To this end, doctors and other members of the geriatric centers will meet in 10 focus groups of 10 participants each. They will discuss exploratory questions about the needs and sensitivity to delirium and POCD, the knowledge about delirium, dementia, and depression, and other aspects of the study. To ensure the implementation of the results, they will be disseminated through workshops: at a training course; at the annual update on dementia (in cooperation with the medical association of Baden-Württemberg); and at the day of general medicine of the Institute of General Medicine and Interprofessional Care in Tübingen. A main result of the project will be a care pathway including information tools (e.g., flyers) and training modules for doctors and doctors’ assistants.

#### Ethics

This is an intervention study in which existing guidelines and recommendations will be implemented, including the S3 guideline of the German Society for Anesthesiology and Intensive Care Medicine (Deutsche Gesellschaft für Anästhesie und Intensivmedizin (DGAI)), the S3 guideline of the Working Group in Geriatric Traumatology (Arbeitsgemeinschaft Alterstraumatologie (AG Alterstraumatologie)) of the German Association of Trauma Surgery (Deutsche Gesellschaft für Unfallchirurgie (DGU)), the guidelines of the National Institute for Clinical Excellence (NICE), the guidelines of the American Geriatrics Society (AGS), and the guidelines of the Difficult Airway Society (DAS). Ethics approval according to the occupational regulations (Berufsordnung (BO)) has been obtained for all centers. All relevant changes will be communicated to the scientific advisory board, the Regional Ethics and Data Monitoring Board (REDMB), the relevant institutional review boards (IRBs), and the clinical trial registry. Given that there are no extra risks, the usual health insurance or civil liability will cover the risks of the trial, and there will be no additional medical liability insurance for the study. For the additional outpatient assessments, patients and their relatives will be covered by travel insurance.

In the preadmission assessments, it is to be expected that approximately 40% of the examined patients will have slight or severe cognitive impairment [72]. However, this does not necessarily restrict their capacity to consent or their legal capacity. Following Kim and Caine [73], we consider that this only occurs when transitioning to a more severe dementia (MMSE < 19). For those patients, the possible damage would be the self-stigmatization as a dementia-endangered person, but the benefit of the intervention would prevail because of its stipulated reduction of delirium and POCD.

On the other hand, if a previously unrecognized severe cognitive disorder was found, the patient probably could not consent, requiring consent from a substitute decision-maker.

## Discussion

This study has several strong features. The study will determine the perioperative delirium prevalence, measured with I-CAM-S, for the first time in a large multicenter German sample. Despite its high prevalence and consequences, delirium is often underdiagnosed in hospitalized older adults. In particular, this is the case for hypoactive delirium because patients with this syndrome are often not disruptive [8]. To tackle this issue, we will use the five-item I-CAM, a useful diagnostic and screening tool for ICD-10 delirium that includes abnormal psychomotor activity and is sensitive for the detection of hypoactive delirium [28].

In addition, we will use a complementary chart review [31] to ascertain the detection of delirium beyond the established sensitivity and specificity of the I-CAM. While we cannot exclude disruptions of blinding of outcome assessors in this complex trial, we nevertheless attempted to ensure blinding of the assessors as much as was feasible in this trial.

Another advantage of the study is the development of a multisector, individualized, multiprofessional and multimodal delirium and POCD prevention program. There is now strong evidence indicating that such multicomponent interventions can prevent delirium in hospitalized patients, and indicating the importance of adequate training of the involved staff [16].

Another strength of the study is the design as a stepped-wedge cluster randomized controlled trial, which allows modeling of effects within and between sites of the delirium prevention and management program. Such evidence is of higher quality than results obtained from nonrandomized studies [74]. In addition, by using a stepped-wedge design, the intervention will be made available to all clusters by the end of the trial [75], avoiding the controversial situation in which control groups have no intervention.

The study will also investigate whether the intervention is cost-effective, so that the improvement of quality of life does not involve higher costs, and the care needs are lower than without the intervention. Given that delirium is highly multifactorial and is linked to many other common geriatric syndromes, it is expected that improving its diagnosis and treatment will be a very practical and effective strategy to improve outcomes, decrease costs, and raise the quality of the healthcare system wide [14].

Finally, the results of the study are intended to be a milestone for new German guidelines for the prevention and management of delirium in surgery, and for dealing with the frequent and insufficiently diagnosed POCD. In this way, the results of an elective operation will be better, the patient safety and quality of life will be improved, and the long-term risk of dementia will be minimized. Furthermore, from an economic perspective, diagnosing cognitive deficits early and minimizing POD and POCD should be beneficial for patients, caregivers, hospitals, and healthcare insurances.

### Trial status

Recruitment commenced in November 2017.

## Additional file

Additional file 1: SPIRIT checklist: recommended items to address in a clinical trial protocol and related documents. (DOCX 56 kb)

## Abbreviations

AE: Adverse event
AG: Arbeitsgemeinschaft (working group)
AGS: American Geriatrics Society
AOK: Allgemeine Ortskrankenkasse (public health insurance company)
ASA: American Society of Anesthesiologists Physical Status classification
BO: Berufsordnung (Professional Code of Conduct)
CAM-S: CAM-based scoring system for assessing delirium severity
CCI: Charlson’s comorbidity index
CHA_2_DS_2_-VASc: Congestive heart failure/left ventricular dysfunction, Hypertension, Age > 75, Diabetes, Stroke/transient ischemic attack/thromboembolism, Vascular disease, Age 65–74, Sex category
CHOPs: Care of Confused Hospitalised Older Persons
CSHAClinical Frailty Scale: Clinical Frailty Scale of the Canadian Study of Health and Aging
DAS: Difficult Airway Society
DGAI: Deutsche Gesellschaft für Anästhesie und Intensivmedizin (German Society for Anesthesiology and Intensive Care Medicine)
DGU: Deutsche Gesellschaft für Unfallchirurgie (German Association of Trauma Surgery)
eCRF: Electronic Case Report Form
EDC: Electronic data capture
EQ-5D-5 L: EuroQol five dimensions questionnaire
G-BA: Gemeinsame Bundesausschuss (Federal Joint Committee)
GC: Geriatric-Check
GPS: Good practice secondary data analysis
G-ZBI: German Zarit Burden interview
HELP: Hospital Elder Life Program
I-CAM: I-Confusion Assessment Method
ICMJE: International Committee of Medical Journal Editors
IQCODE: Informant Questionnaire on Cognitive Decline in the Elderly
IRBs: Institutional review boards
MAR: Missing at random
MMSE: Mini Mental State Examination
MNA-SF: Mini Nutritional Assessment Short Form
MoCA: Montreal Cognitive Assessment
NICE: National Institute for Clinical Excellence
NOSGER II: Nurses’ Observation Scale for Geriatric Patients
NRS Pain: Numerical Rating Scale of Pain
NuDESC: Nursing Delirium Screening Scale
POCD: Postoperative cognitive dysfunction
POD: Postoperative delirium
PSQI-Basic: Pittsburgh Sleep Quality Index—Basic
QALY: Quality-adjusted life year
RASS: Richmond Agitation–Sedation Scale
REDMB: Regional Ethics and Data Monitoring Board
ROC: Receiver operating characteristics
SAE: Serious adverse event
SF-12: 12-Item Short Form Survey
SMI: Subjective memory impairment
SPIRIT: Standard Protocol Items: Recommendations for Interventional Trials
Stop-BANG: STOP-BANG questionnaire
TAU: Treatment as usual
TMT A and B: Trail Making Test A and B
TUG: Timed Up and Go
